# Risk factors and predicting nomogram for the clinical deterioration of non-severe community-acquired pneumonia

**DOI:** 10.1186/s12890-023-02813-w

**Published:** 2024-01-27

**Authors:** Cheng-bin Xu, Shan-shan Su, Jia Yu, Xiong Lei, Peng-cheng Lin, Qing Wu, Ying Zhou, Yu-ping Li

**Affiliations:** 1 The Key Laboratory of Interventional Pulmonology of Zhejiang Province, Department of Pulmonary and Critical Care Medicine, The First Affiliated Hospital of Wenzhou Medical University, South Baixiang, Ouhai District, Wenzhou, Zhejiang Province 325015 People’s Republic of China; 2 The Center of Laboratory and Diagnosis, The First Affiliated Hospital of Wenzhou Medical University, Wenzhou, Zhejiang Province 325015 People’s Republic of China

**Keywords:** Risk factors, Nomogram, Clinical deterioration, Non-severe community acquired pneumonia

## Abstract

**Background:**

Currently, there remains insufficient focus on non-severe community-acquired pneumonia (CAP) patients who are at risk of clinical deterioration, and there is also a dearth of research on the related risk factors. Early recognition of hospitalized patients at risk of clinical deterioration will be beneficial for their clinical management.

**Method:**

A retrospective study was conducted in The First Affiliated Hospital of Wenzhou Medical University, China, spanning from January 1, 2018 to April 30, 2022, and involving a total of 1,632 non-severe CAP patients. Based on whether their condition worsened within 72 h of admission, patients were divided into a clinical deterioration group and a non-clinical deterioration group. Additionally, all patients were randomly assigned to a training set containing 75% of patients and a validation set containing 25% of patients. In the training set, risk factors for clinical deterioration in patients with non-severe CAP were identified by using LASSO regression analysis and multivariate logistic regression analysis. A nomogram was developed based on identified risk factors. The effectiveness of the nomogram in both the training and validation sets was assessed using Receiver Operating Characteristic (ROC) curves, calibration curves, and decision curve analysis (DCA).

**Results:**

Age, body mass index (BMI), body temperature, cardiovascular comorbidity, respiratory rate, LDH level, lymphocyte count and D-dimer level were identified as risk factors associated with the clinical deterioration of non-severe CAP within 72 h of admission. The area under curve (AUC) value of the nomogram was 0.78 (95% CI: 0.74–0.82) in the training set and 0.75 (95% CI: 0.67–0.83) in the validation set. Furthermore, the calibration curves for both the training and validation sets indicated that the predicted probability of clinical deterioration aligned with the actual probability. Additionally, DCA revealed clinical utility for the nomogram at a specific threshold probability.

**Conclusion:**

The study successfully identified the risk factors linked to the clinical deterioration of non-severe CAP and constructed a nomogram for predicting the probability of deterioration. The nomogram demonstrated favorable predictive performance and has the potential to aid in the early identification and management of non-severe CAP patients at elevated risk of deterioration.

**Supplementary Information:**

The online version contains supplementary material available at 10.1186/s12890-023-02813-w.

## Introduction

Community-acquired pneumonia (CAP), a global acute disease caused by various pathogenic microorganisms, including bacteria, fungi, viruses, etc., resulting in significant mortality and hospitalizations annually [1]. A 2-year investigation in the USA revealed an age-adjusted incidence of 649 hospitalizations for CAP per 100,000 adults per year, with a hospitalization mortality rate of 6.5%, equating to 102,821 annual deaths [2]. Early identification of severe CAP patients, as defined by the IDSA/ATS 2007 criteria [3], is essential for guiding Intensive Care Unit (ICU) admission and optimizing in-hospital management. Additionally, CAP severity assessment scores like CURB-65, CRB-65, Pneumonia Severity Index (PSI) scores are commonly utilized to identify high-risk CAP patients [4, 5]. Given the successful use of these severity assessment scores, there is a growing interest among researchers in developing improved scoring systems to predict CAP mortality and identify severe CAP patients.

Academics have highlighted a fundamental issue with existing CAP severity assessment scores intended to predict mortality, noting their limited ability to accurately identify patients requiring more than standard care, and they emphasize the importance of developing a tool for clinical physicians to differentiate patients at risk of deterioration [6]. While immediate changes to therapies may not be necessary for patients who are at risk of deterioration immediate, they do require more intensive monitoring [6]. Notably, reach by Ilg and colleagues revealed that some patients with low CURB-65 scores (≤ 1) still required admission to the ICU (15.6%) or critical care intervention (6.4%) [7]. Furthermore, Li and colleagues emphasized the strong correction between non-severe CAP patients meeting certain IDSA/ATS secondary criteria (confusion, PaO2/FiO2 ≤ 250 mmHg, and uremia) and mortality, suggesting the need to prioritize intensive care and treatment for these individuals [8]. Consequently, there is a need to focus on a specific subset of non-severe CAP patients and explore a tool to identify clinical deterioration in non-severe CAP patients.

Currently, there is a deficiency in the availability of a scoring system or model for forecasting clinical deterioration in non-severe CAP patients. The objective of this study was to identify the risk factors associated with clinical deterioration and construct a nomogram for predicting the likelihood of clinical deterioration in non-severe CAP patients. The assessment of the nomogram’s discriminatory ability was conducted using ROC curves, while calibration curves were employed to assess its accuracy. Additionally, DCA curves were utilized to evaluate the clinical utility of the nomogram.

This study received approval from the Ethics Committee of the First Affiliated Hospital of Wenzhou Medical University (2023-R158) and was conducted following the Declaration of Helsinki (as revised in 2013). No informed consent was required due to the retrospective nature of the study.

## Methods

### Study design

A single-center retrospective cohort study was conducted at the respiratory department of The First Affiliated Hospital of Wenzhou Medical University, a regional medical center in southern Zhejiang Province. From January 1, 2018 to April 30, 2022, a total of 1,632 non-severe CAP cases were collected through the electronic medical record system. To facilitate further investigation, we randomly assigned 75% of cases into the training set (1224 cases) and 25% into the internal validation set (408 cases). In the training set, patients were categorized into a clinical deterioration group and a clinical non-deterioration group based on whether their condition worsened within 72 h of admission to the respiratory department. The study examined the risk factors for clinical deterioration by comparing the differences between the two groups using statistical analysis. Meanwhile, a nomogram was established for forecasting clinical deterioration. The predictive ability of the nomogram was confirmed in the internal validation set.

### Participants

Patients who met the clinical diagnostic criteria for CAP upon admission were included in the study. The specific standards included: (1) Community onset; (2) Chest radiograph suggestive of acute exudation or consolidation; (3) Clinical manifestations associated with pneumonia: (1) Emerging cough or expectoration or worsening of pre-existing respiratory disease symptoms, with or without dyspnea, chest pain, hemoptysis, or purulent sputum; (2) Fever; (3) Peripheral white blood cell count < 4 × 10^9^/L or > 10 × 10^9^/L, with or without a left shift; (4) Signs of solid lung lesions and or wet rales. Patients had to meet criteria 1 and 2, as well as any one of the criteria 3 to be clinically diagnosed [9]. Exclusion criteria included: (1) Patients who were under 18 years old; (2) Patients who were diagnosed with severe CAP according to IDSA/ATS 2007 criteria at admission; (3) Patients had been hospitalized for over 48 h; (4) Patients were pregnant or immunocompromised; (5) Patients with significant missing data. The definition of an immunocompromised state was based on previous research [10]. Notably, patients with COVID-19 were not part of the study.

### Definition of clinical deterioration

Currently, there is no single definition for clinical deterioration in patients with CAP. Based on previous researches and the specific patient cases in hospital, we regarded patients who met any of the following criteria within 72 h of admission as clinically deteriorating: (1) Elevated level of care; (2) Increased oxygen demand; (3) Chest imaging suggests progression; (4) Added need for renal replacement therapy; (5) Application of vasoactive drugs; (6) Death or automatic discharge. It is important to note that general hospital wards commonly provide two levels of care: first-class nursing and second-class nursing. Patients who are provided with first-class nursing care exhibit unstable medical conditions that may change unpredictably, while those receiving second-class nursing care have stable medical conditions.

### Data collection

We collected clinical data from non-severe CAP patients within 24 h of admission to the respiratory department through the electronic case system, including demographic data, comorbidities, index of laboratory examination, chest imaging features, duration of antibiotic use before admission, and time interval between onset and admission. Table 1 shows detailed data.

**Table 1:** Baseline of all patients with non-severe CAP

Characteristics	Total (n = 1632)	Non-Clinical Deterioration (n = 1491)	Clinical Deterioration (n = 141)	*P* value
Sex, female n (%)	675 (41)	626 (42)	49 (35)	0.115
Age(year), Median (Q1, Q3)^a^	58 (45, 69)	57 (44, 68)	67 (54, 76)	< 0.001
BMI(Kg/m^2^), Median (Q1, Q3)	22.43(20.42, 24.92)	22.39(20.42, 24.81)	23.1(20.76, 26.44)	0.036
Hypertension, n (%)	495 (30)	441 (30)	54 (38)	0.04
Diabetes, n (%)	210 (13)	187 (13)	23 (16)	0.252
Cardiovascular disease^c^, n (%)	155 (9)	127 (9)	28 (20)	< 0.001
Liver disease, n (%)	133 (8)	124 (8)	9 (6)	0.521
Gastrointestinal diseases, n (%)	71 (4)	67 (4)	4 (3)	0.48
Chronic kidney disease, n (%)	29 (2)	26 (2)	3 (2)	0.734
Cerebrovascular disease, n (%)	66 (4)	55 (4)	11 (8)	0.032
Basic lung disease^b^, n (%)	78 (5)	67 (4)	11 (8)	0.12
Pleural effusion, n (%)	441 (27)	385 (26)	56 (40)	< 0.001
Bilateral lung lesions, n (%)	759 (47)	679 (46)	80 (57)	0.014
Time interval between onset and admission(day), Median (Q1, Q3)	7 (4, 10)	7 (4, 10)	6 (4, 10)	0.448
Duration of antibiotic use before admission(day), Median (Q1, Q3)	2.5 (1, 4)	3 (1, 4)	2 (1, 4)	0.545
Body temperature(℃), Median (Q1, Q3)	37.1(36.7, 37.8)	37.1 (36.7, 37.7)	37.5(36.9, 38.5)	< 0.001
Systolic pressure(mmHg), Median (Q1, Q3)	123(111, 138)	123 (110, 137)	128(115, 141)	0.014
Diastolic pressure(mmHg), Median (Q1, Q3)	76 (69, 84)	76 (69, 84)	76 (67, 84)	0.297
Respiratory rate(/min), Median (Q1, Q3)	20 (20, 20)	20 (20, 20)	20 (20, 20)	< 0.001
Pulse(/min), Median (Q1, Q3)	88 (78, 100)	88 (77, 99)	91 (80, 103)	0.005
SpO_2_(%), Median (Q1, Q3)	97 (95, 98)	97 (95, 98)	96 (94, 97)	< 0.001
WBC(×10^9^/L), Median (Q1, Q3)	7.16(5.48, 9.89)	7.06 (5.46, 9.7)	8.72(5.82, 11.99)	< 0.001
ANC(×10^9^/L), Median (Q1, Q3)	5.04(3.46, 7.6)	4.91 (3.4, 7.37)	6.54(4.21, 9.62)	< 0.001
LYM(×10^9^/L), Median (Q1, Q3)	1.3(0.96, 1.72)	1.33 (0.98, 1.75)	1.06(0.74, 1.43)	< 0.001
RBC(×10^12^/L), Median (Q1, Q3)	4.16(3.8, 4.55)	4.17 (3.81, 4.56)	4.12(3.74, 4.48)	0.06
HB(g/L), Median (Q1, Q3)	126(114, 138)	126 (115, 138)	123 (111, 136)	0.023
PLT(×10^9^/L), Median (Q1, Q3)	241(184, 307)	242 (186, 307)	228 (170, 312)	0.22
CRP(mg/L), Median (Q1, Q3)	61.15(19.9, 114.78)	56.9 (17.7, 110.36)	102(56.3, 173)	< 0.001
PCT(ng/ml, Median (Q1, Q3)	0.09(0.04, 0.29)	0.08 (0.04, 0.25)	0.17(0.08, 0.73)	< 0.001
TBIL(µmol/L), Median (Q1, Q3)	8 (6, 12)	8 (6, 12)	10 (7, 15)	< 0.001
ALT(U/L), Median (Q1, Q3)	23(14, 44.25)	23 (14, 43)	26 (16, 49)	0.067
AST(U/L), Median (Q1, Q3)	25 (19, 39)	24 (18, 38)	35 (22, 53)	< 0.001
ALB(g/L), Mean ± SD	36.23 ± 5.4	36.54 ± 5.34	32.9 ± 4.92	< 0.001
BUN(mmol/L), Median (Q1, Q3)	4.6 (3.6, 6)	4.6 (3.6, 5.9)	5 (3.7, 6.9)	0.02
Cr(µmol/L), Median (Q1, Q3)	66 (55, 80)	65 (55, 80)	72 (55, 86)	0.038
eGFR, Median (Q1, Q3)	100.2(85.68, 113.8)	101.1(87.05, 114.15)	92.6(77.1, 107.9)	< 0.001
UA(µmol/L), Median (Q1, Q3)	252.5(193, 316)	253 (195.5, 315.5)	228 (161, 325)	0.031
K^+^(mmol/L), Median (Q1, Q3)	3.75(3.49, 4.01)	3.76 (3.5, 4.01)	3.74(3.42, 4.01)	0.229
Na^+^(mmol/L), Median (Q1, Q3)	139(136.75, 141)	139 (137, 141)	138(135, 140)	< 0.001
CL^−^(mmol/L), Median (Q1, Q3)	103(100, 105)	103 (100, 105)	102 (99, 104)	0.002
TC(mmol/L), Median (Q1, Q3)	4.11(3.51, 4.79)	4.15 (3.56, 4.81)	3.73 (3.1, 4.47)	< 0.001
TG(mmol/L), Median (Q1, Q3)	1.08(0.82, 1.49)	1.08 (0.82, 1.5)	1.03(0.85, 1.38)	0.237
HDL(mmol/L), Median (Q1, Q3)	0.92(0.74, 1.14)	0.93 (0.75, 1.15)	0.86(0.65, 1.05)	< 0.001
LDL(mmol/L), Median (Q1, Q3)	2.4(1.91, 2.92)	2.43 (1.93, 2.96)	2.06(1.6, 2.52)	< 0.001
CK(U/L), Median (Q1, Q3)	71 (47, 113)	70 (48, 111)	84 (44, 158)	0.061
LDH(U/L), Median (Q1, Q3)	213(178, 256)	210 (176, 252)	248(202, 320)	< 0.001
D-Dimer(mg/L), Median (Q1, Q3)	0.75(0.39, 1.54)	0.71 (0.38, 1.46)	1.33(0.81, 2.12)	< 0.001

^a^: Q1, first quartile; Q3, third Quartile. ^b^: basic lung diseases including chronic bronchi, asthma, chronic obstructive pulmonary disease, silicosis in study. ^c^: cardiovascular disease including coronary atherosclerotic heart disease and atrial fibrillation. WBC: white blood cell. ANC: absolute neutrophil value. LYM: absolute lymphocyte value. HB: hemoglobin. CRP: C-reactive protein. PCT: procalcitonin. TBIL: total bilirubin. ALT: glutathione aminotransferase. AST: glutathione transaminase. ALB: albumin. BUN: Urea. Cr: creatinine. UA: uric acid. TC: total cholesterol. TG: triglycerides. HDL: high-density cholesterol. LDL: low-density cholesterol. CK: creatives kinase. LDH: lactate dehydrogenase

### Statistical analysis

Normally distributed measurement variables were presented as mean ± standard deviation (SD), and measurement variables that were not normally distributed were presented as median (first quartile and third quartile (Q1, Q3)), whereas categorical variables were presented as count (percentage). T test and Wilcoxon rank sum test were used for normally and abnormally distributed measurement variables, respectively. For categorical variables, the chi square test or Fisher exact test was used. Variables with missing values > 20% were removed and multiple imputation was used to fill in the missing values. In the training set, LASSO regression was used to filter variables, which were then incorporated into logistic regression to identify risk factors and establish a risk prediction model. The predictive ability of the model was tested in the internal validation group. R software (version 4.2.3) was used to do statistical analysis. Statistical significance was considered if the two-tailed *P* value was < 0.05.

## Results

### Baseline of patients

A total of 1632 non-severe CAP patients were included in this study, with a median age of 58 years (IQR: 45–69 years), 957 patients were male (59%), median BMI was 22.43 kg/m^2^ (IQR: 20.42–24.92 kg/m^2^), median body temperature was 37.1 ℃(IQR: 36.7–37.8 ℃), median respiratory rate was 20/min (IQR: 20–20 /min), median systolic pressure was 123 mmHg (IQR: 111–138 mmHg), median diastolic pressure was 76 mmHg (IQR: 69–84 mmHg), median pulse was 88/min (IQR:78–100 /min), median duration of antibiotic use before admission was 2.5 days (IQR: 1–4 days), median time interval between onset and admission was 7 days (IQR: 4–10 days). Hypertension was the most common comorbidity with 495 patients (30%), followed by diabetes with 210 patients (13%), and chronic kidney disease was the least common comorbidity with 29 patients (2%). Chest imaging suggested pleural effusion in 441 patients (27%) and bilateral lung lesions in 759 patients (47%). A total of 141 patients experienced clinical deterioration and 1491 patients did not. Age, BMI, comorbidities (hypertension, cardiovascular disease, cerebrovascular disease), pleural effusion, bilateral lung lesions, body temperature, systolic pressure, respiratory rate, pulse, SpO_2_, WBC count, ANC count, LYM count, HB, CRP, PCT, TBIL, AST, ALB, BUN, Cr, eGFR, UA, Na^+^, Cl^−^, TC, HDL, LDL, LDH, and D-Dimer were discovered to have statistical differences between the clinical deterioration group and non-clinical deterioration group (*P* < 0.05) (Table 1).

### Risk factors and model of clinical deterioration

**Fig. 1:**
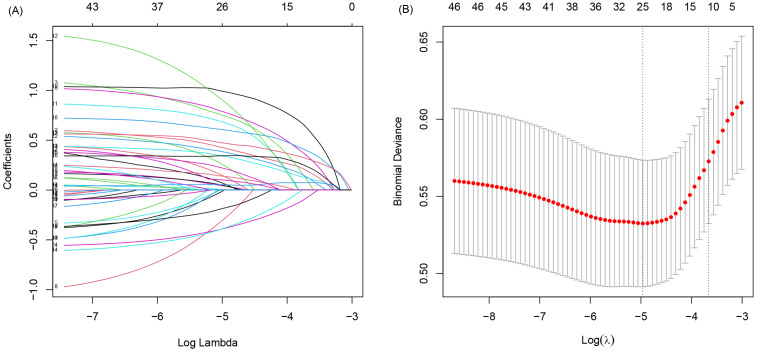
Filtering variables for logistic regression by using LASSO regression. (**A**) Model coefficient trendlines of the total 47 variables for clinical deterioration. The y-axis represents the coefficient of the variable, the lower x-axis indicates the log (λ), the upper x-axis indicates the number of variables. As log (λ) increases, the coefficient of variables tends to zero, and the number of corresponding variables gradually decreases. (**B**) Cross-validation curves for LASSO regression. The y-axis represents binomial deviation. The dashed line on the left shows the log (λ) when the model deviation is minimal, and the dashed line on the right shows the log (λ) when the model deviation is minimal plus one standard error. We selected the log (λ) and the variables corresponding to the dashed line on the right

**Fig. 2:**
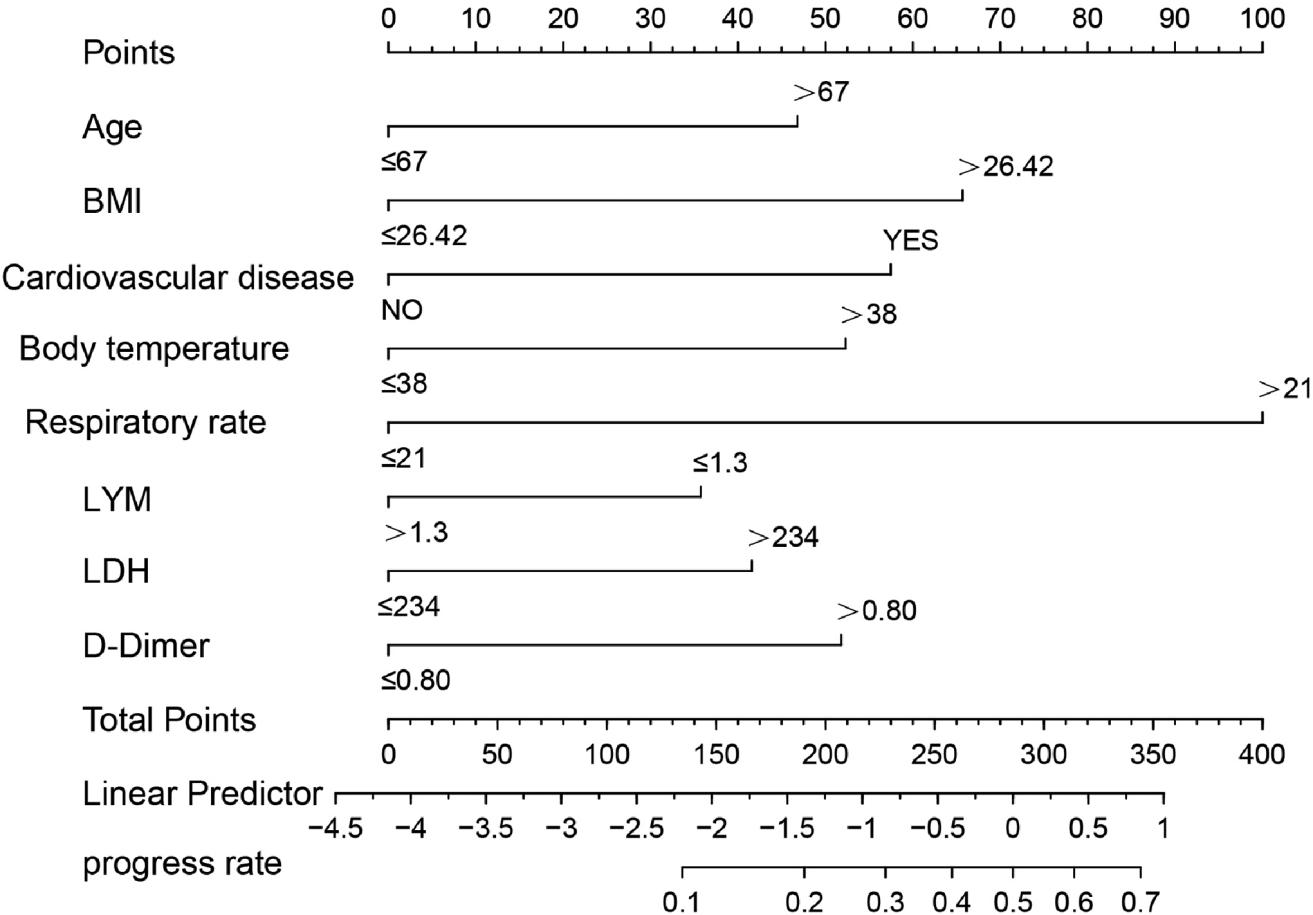
Nomogram for risk prediction for clinical deterioration of non-severe CAP.

To further analyze the data, 36 continuous variables were transformed into categorical variables using cutoff or median values (see Additional file 1). All patients were randomly divided into two sets, with 75% in the training set (Table 2) and 25% in the validation set. In the training set, LASSO regression analysis was used to screen all 47 variables, result in the selection of 11 variables for multivariate logistic regression analysis (Fig. 1). Ultimately, eight variables including age, BMI, combined cardiovascular disease, body temperature, respiratory rate, LYM count, LDH, and D-Dimer were identified as risk factors for the clinical deterioration of non-severe CAP (Table 3). Figure 2 shows the nomogram constructed to predict the risk of clinical deterioration in non-severe CAP patients.

**Table 2:** Clinical characteristics of non-clinical deterioration and clinical deterioration patients in training set based on cutoff or median values

Characteristics	Total (n = 1224)	Non-Clinical Deterioration (n = 1113)	Clinical Deterioration (n = 111)	*P* value
Sex, female n (%)	502 (41)	465 (42)	37 (33)	0.104
Age >67(year), n (%)	326 (27)	275 (25)	51 (46)	< 0.001
BMI >26.42 (kg/m^2^), n (%)	163 (13)	137 (12)	26 (23)	0.002
Hypertension, n (%)	367 (30)	324 (29)	43 (39)	0.045
Diabetes, n (%)	159 (13)	143 (13)	16 (14)	0.749
Cardiovascular disease^c^, n (%)	121 (10)	96 (9)	25 (23)	< 0.001
Liver disease, n (%)	93 (8)	88 (8)	5 (5)	0.27
Gastrointestinal diseases, n (%)	53 (4)	51 (5)	2 (2)	0.223
Chronic kidney disease, n (%)	20 (2)	19 (2)	1 (1)	1
Cerebrovascular disease, n (%)	52 (4)	45 (4)	7 (6)	0.317
Basic lung disease^b^, n (%)	57 (5)	46 (4)	11 (10)	0.012
Pleural effusion, n (%)	330 (27)	285 (26)	45 (41)	0.001
Bilateral lung lesions, n (%)	565 (46)	504 (45)	61 (55)	0.064
Time interval between onset and admission >2 (day), n (%)	1109 (91)	1007 (90)	102 (92)	0.751
Duration of antibiotic use before admission >7 (day), n (%)	63 (5)	55 (5)	8 (7)	0.421
Body temperature >38 (℃), n (%)	224 (18)	181 (16)	43 (39)	< 0.001
Systolic pressure>133 (mmHg), n (%)	390 (32)	341 (31)	49 (44)	0.005
Diastolic pressure >84 (mmHg), n (%)	279 (23)	251 (23)	28 (25)	0.602
Respiratory rate >21 (/min), n (%)	31 (3)	20 (2)	11 (10)	< 0.001
Pulse >95 (/min), n (%)	391 (32)	344 (31)	47 (42)	0.018
SpO_2_ >97 (%), n (%)	369 (30)	341 (31)	28 (25)	0.282
WBC >9.86 (×10^9^/L), n (%)	298 (24)	250 (22)	48 (43)	< 0.001
ANC >6.35 (×10^9^/L), n (%)	416 (34)	354 (32)	62 (56)	< 0.001
LYM >1.3 (×10^9^/L), n (%)	609 (50)	577 (52)	32 (29)	< 0.001
RBC >4.16 (×10^12^/L), n (%)	613 (50)	560 (50)	53 (48)	0.677
HB >126 (g/L), n (%)	598 (49)	547 (49)	51 (46)	0.587
PLT >366 (×10^9^/L), n (%)	141 (12)	125 (11)	16 (14)	0.398
CRP >82.7 (mg/L), n (%)	489 (40)	417 (37)	72 (65)	< 0.001
PCT >0.078 (ng/ml), n (%)	661 (54)	576 (52)	85 (77)	< 0.001
TBIL >9 (µmol/L), n (%)	505 (41)	442 (40)	63 (57)	< 0.001
ALT >35 (U/L), n (%)	379 (31)	339 (30)	40 (36)	0.269
AST >35 (U/L), n (%)	359 (29)	308 (28)	51 (46)	< 0.001
ALB >36 (g/L), n (%)	625 (51)	589 (53)	36 (32)	< 0.001
BUN >6.6 (mmol/L), n (%)	209 (17)	176 (16)	33 (30)	< 0.001
Cr >74 (µmol/L), n (%)	418 (34)	364 (33)	54 (49)	0.001
eGFR >100.2, n (%)	615 (50)	577 (52)	38 (34)	< 0.001
UA >367 (µmol/L), n (%)	148 (12)	132 (12)	16 (14)	0.526
K^+^ >4.41 (mmol/L), n (%)	63 (5)	55 (5)	8 (7)	0.421
Na^+^ >139 (mmol/L), n (%)	502 (41)	472 (42)	30 (27)	0.002
CL^−^ >103 (mmol/L), n (%)	524 (43)	490 (44)	34 (31)	0.009
TC >2.20 (mmol/L), n (%)	1206 (99)	1098 (99)	108 (97)	0.219
TG >0.65 (mmol/L), n (%)	1100 (90)	993 (89)	107 (96)	0.026
HDL >0.92 (mmol/L), n (%)	617 (50)	572 (51)	45 (41)	0.037
LDL >2.4 (mmol/L), n (%)	613 (50)	578 (52)	35 (32)	< 0.001
CK >120 (U/L), n (%)	275 (22)	238 (21)	37 (33)	0.006
LDH >234 (U/L), n (%)	431 (35)	366 (33)	65 (59)	< 0.001
D-Dimer >0.80 (mg/L), n (%)	583 (48)	500 (45)	83 (75)	< 0.001

^b^: basic lung diseases including chronic bronchi, asthma, chronic obstructive pulmonary disease, silicosis in study. ^c^: cardiovascular disease including coronary atherosclerotic heart disease and atrial fibrillation. WBC: white blood cell. ANC: absolute neutrophil value. LYM: absolute lymphocyte value. HB: hemoglobin. CRP: C-reactive protein. PCT: procalcitonin. TBIL: total bilirubin. ALT: glutathione aminotransferase. AST: glutathione transaminase. ALB: albumin. BUN: Urea. Cr: creatinine. UA: uric acid. TC: total cholesterol. TG: triglycerides. HDL: high-density cholesterol. LDL: low-density cholesterol. CK: creatine kinase. LDH: lactate dehydrogenase

**Table 3:** The risk factors for clinical deterioration in multivariate logistic regression analysis among non-severe CAP patients in training set

Variable	*Β*	*Z*	*P*	Odds ratio	95% CI
Intercept	-3.6300	-13.17	< 0.0001		
Age > 67(year)	0.6791	2.91	0.0036	1.97	1.25–3.11
BMI >26.42(kg/m2)	0.9531	3.55	0.0004	2.59	1.51–4.35
Cardiovascular disease^c^	0.8340	2.92	0.0035	2.30	1.30–3.99
Body temperature> 38(℃)	0.7591	3.23	0.0012	2.14	1.34–3.37
Respiratory rate> 21(/min)	1.4512	3.40	0.0007	4.27	1.80–9.72
LYM ≤ 1.3(×10^9^/L)	0.5186	2.17	0.0297	1.68	1.06–2.71
LDH> 234(U/L)	0.6033	2.68	0.0074	1.83	1.18–2.85
D-Dimer >0.80(mg/L)	0.7519	3.00	0.0027	2.12	1.31–3.51

^c^: cardiovascular disease including coronary atherosclerotic heart disease and atrial fibrillation. LYM: absolute lymphocyte value. LDH: lactate dehydrogenase

### Model verification

**Fig. 3:**
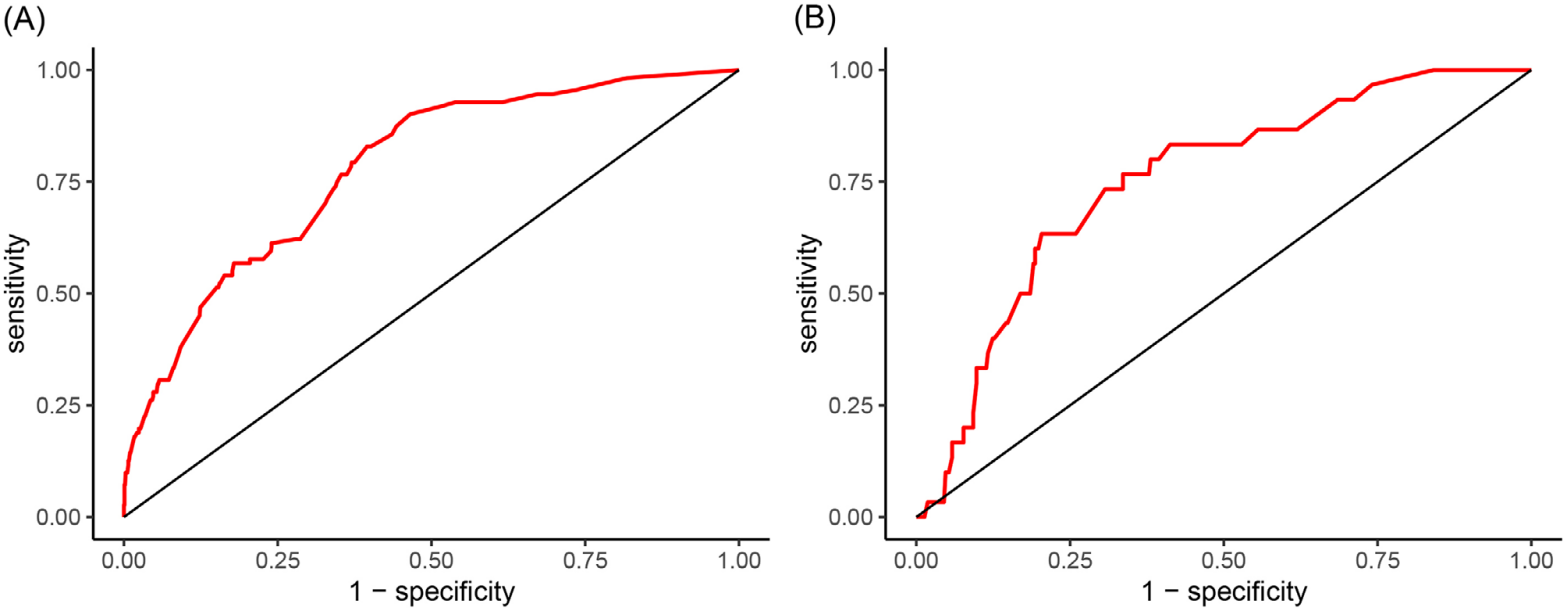
ROC of the nomogram for non-severe CAP progression risk prediction in the training set (**A**) and validation set (**B**)

**Fig. 4:**
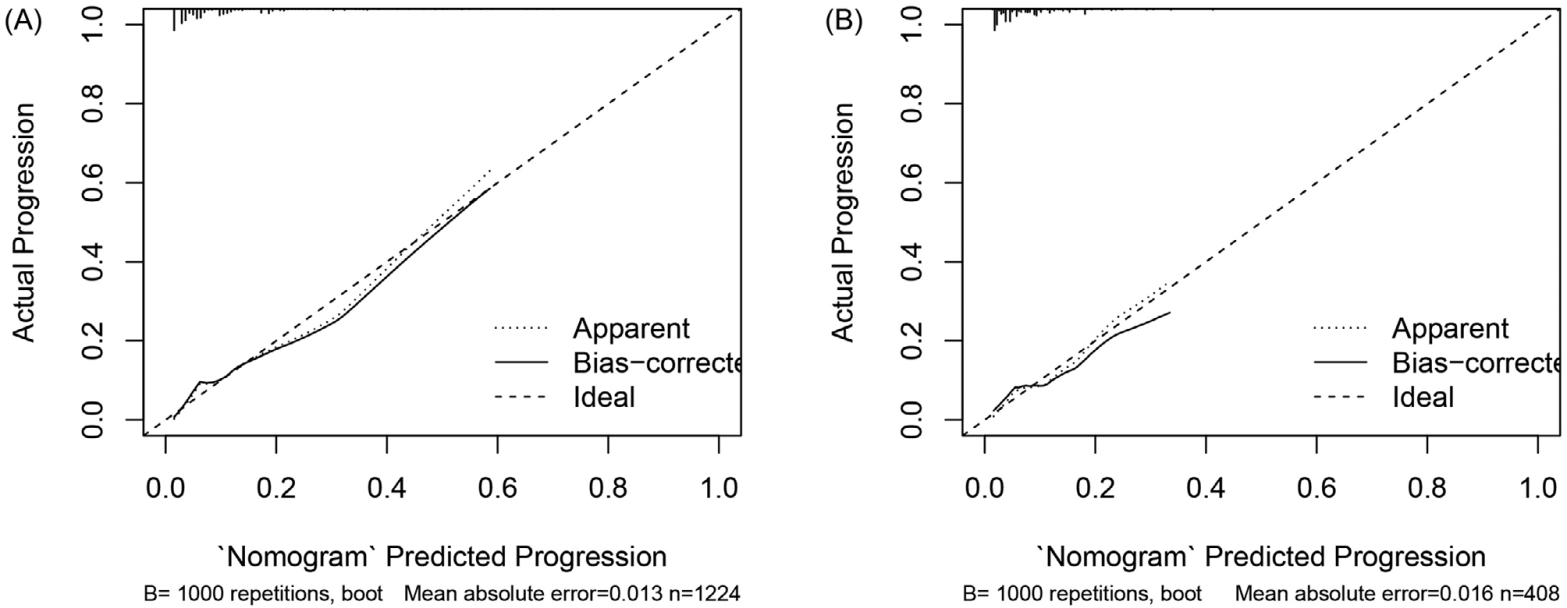
Calibration curve of the nomogram for non-severe CAP progression risk prediction in the training set (**A**) and in the validation set (**B**) separately. The X-axis indicates the probability of clinical deterioration in non-severe CAP patients predicted by the nomogram, and the y-axis indicates the probability of clinical deterioration in CAP patients actually occurring. The dotted line represents the ideal performance, and the solid line represents the performance of the nomogram. The closer to the dotted line, the better the prediction effect

**Fig. 5:**
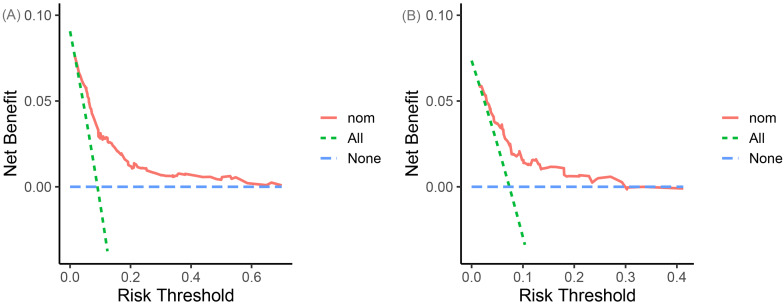
Decision curve analysis (DCA) for the nomogram of clinical deterioration in the training set (**A**) and validation set (**B**). The X-axis represents the threshold probability. The y-axis represents net benefit. The green dashed line indicates intervention for all, and the blue dashed line indicates intervention for none. The red solid line indicates intervention for the nomogram

To demonstrate the accuracy of the nomogram in forecasting the likelihood of clinical deterioration in non-severe CAP patients, separate AUC curves were generated for the training and validation sets (Fig. 3). The findings revealed that the AUC of the nomogram in the training set reached 0.78 (95% CI: 0.74–0.82), and in the validation set, the AUC was 0.75 (95% CI: 0.67–0.83), indicating that the nomogram had an acceptable ability to predict risk. The calibration curve of the nomogram was utilized to access the consistency between model prediction probability and observation probability, and it performed well in both the training and validation sets (Fig. 4). The DCAs in the training and validation sets demonstrated the risk prediction nomogram had a net clinical benefit at a certain threshold probability (Fig. 5).

## Discussion

CAP is a highly prevalent and potentially fatal infectious disease [11] that has garnered significant attention from researchers and physicians. There is a growing interest in identifying high-risk patients and effectively managing those who are critically ill. Current research indicates dissatisfaction with the use of pneumonia severity scoring systems for predicting mortality. Kolditz et al. discovered that copeptin, the C-terminal peptide of the precursor protein to AVP, effectively predicted persistent clinical instability after 72 h in CAP patients with an AUC of 0.74 [12]. A reach team in Taiwan revealed six clinical factors, including age, pleural effusion, hemoglobin, WBC count, respiratory rate, and duration of defervescence, were associated with the progression of CAP in hospitalized children [13]. In recent years, due to the outbreak and prevalence of COVID-19, many studies have been conducted to identify the risk factors or prediction models for the deterioration of COVID-19 [14–17]. However, these studies lacked a standardized definition of worsening condition and frequently observed patients throughout their entire hospital stay, despite using indicators recorded upon admission or within 24 h of admission. Currently, there are few studies that predict clinical deterioration in non-severe CAP patients within 72 h of admission, despite some scholars noting that a part of patients with low CURB-65 scores received intensive care Interventions [7]. Therefore, a retrospective study was conducted, revealing that several factors including age, BMI, combined cardiovascular disease, body temperature, respiratory rate, LYM count, LDH, and D-Dimer were associated with the clinical deterioration of non-severe CAP patients. Additionally, a nomogram was developed to forecast the risk of clinical deterioration, facilitating the identification of patients requiring increased attention within 72 h of admission.

Currently, numerous studies have identified the risk factors associated with the incidence, severity, and prognosis of CAP. Age has been identified as a significant risk factor [18], with research indicating higher hospitalization rates among elderly patients (age ≥ 65 years) compare to non-elderly patients (2093 per 100,000 vs. 327 per 100,000) [2]. Furthermore, nearly 90% of pneumonia-related deaths occurred in patients aged > 65 years [19]. Consistent with these findings, this study also observed that CAP patients aged > 67 years were more prone to deteriorate, possibly due to age-related immunosuppression resulting from medication interactions, comorbidities, and immune system degradation [20]. Notably, the age cutoff value in this study was higher than 65 years due to different endpoints. The relationship between CAP and BMI is intricate. While Bramley and colleagues found a link between being overweight or obese and ICU admission in children, no association was observed between elevated BMI and severe CAP outcomes in adults [21]. In a separate investigation, obese patients (BMI > 30 kg/m^2^) had lower 6-year all-cause mortality in CAP than normal-weight individuals, supporting the phenomenon known as the “obesity paradox” [22]. In this study, non-severe CAP patients with a BMI > 26.42 kg/m^2^ were more likely to experience clinical deterioration. Fever is a prevalent symptom of CAP. According to Huang et al., a prolonged fever lasting > 72 h was identified as a risk factor for the progression of CAP in children [13]. Another study indicated that the absence of fever, indicative of compromised immune function and ineffective host defense, was associated with higher mortality in CAP patients [23]. In this study, immunosuppressed CAP patients were ruled out, and patients with a body temperature > 38℃ were more likely to experience clinical deterioration, possibly due to more severe inflammatory reactions. Furthermore, Huang et al. identified tachypnea, which is a symptom to predict acute respiratory distress, was associated with the development of progressive CAP in children [13]. Similarity, patients with a respiratory rate > 21/min were at an elevated risk of deterioration in this study. Notably, Guo and colleagues conducted a study to update the cut-off values of severity scoring systems for CAP, and the findings indicated that a respiratory rate ≥ 22/min was more effective than a respiratory rate ≥ 30/min in predicting mortality [24].

Comorbidities have been identified as a risk factor for the incidence and poor prognosis of CAP, particularly among the elderly [25]. In this study, various comorbidities were taken into account, with cardiovascular disease emerging as the most prevalent comorbidity alongside hypertension and diabetes. There was an association between cardiovascular disease and deterioration in non-severe CAP patients in study, underscoring the need for heightened attention to CAP patients with cardiovascular disease, even in the absence of severe initial symptoms. There exists an interaction between CAP and cardiovascular disease, with reports indicating that CAP is linked to increased likelihood of acute coronary syndrome (ACS) (OR: 3.02) and all cardiovascular diseases (OR: 3.37) [26]. The precise mechanism through which CAP exacerbates or triggers cardiovascular disease remain incompletely understood, although Inflammation, endothelial dysfunction, and myocardial damage are discovered to play pivotal roles [26]. Furthermore, CAP patients with cardiovascular disease are at heightened risk of hospitalization and in-hospital mortality. In Spain, the hospitalization rate for CAP patients with cardiovascular disease aged ≥ 60 years was reported to reach 55.27 hospitalizations per 100,000 inhabitants, and in-hospital mortality rate reach to 32.71 deaths per 100,000 inhabitants [27]. Another study demonstrated that elderly severe CAP patients with cardiovascular disease had a significantly higher risk of in-hospital mortality than patients without cardiovascular disease (53.3 vs. 33.8%) [28].

LYM ≤ 1.3 × 10^9^ /L was discovered to be a risk factor for the clinical deterioration of non-severe CAP in this study, indicating the significance of focusing on patients with low LYM or lymphopenia. Lymphopenia may signify an imbalance in the host’s immune response to infection, leading to a relatively immunosuppressive state for patients [29]. Cilloniz et al. reported a specific immunophenotype of CAP, termed lymphopenic (< 724 lymphocytes/mm^3^) CAP (L-CAP), characterized by a decrease in CD4 + T lymphocytes, an increased inflammatory response, and a poorer prognosis [29]. However, due to missing data, we were unable to analyze cytokines and lymphocyte subsets in patients. Previous studies have demonstrated that a low lymphocyte count (1–2 × 10^9^/L) is associated with increased short-term and long-term mortality, and lymphopenia has been identified as a risk factor for the severity and poor outcomes in patients with COVID-19 [30, 31]. Serum LDH > 234 U/L was identified as another risk factor for the development of progressive CAP. Previous literature suggested that serum LDH was associated with severity and poor prognosis in patients with COVID-19 [32–34]. However, it was reported that LDH may not be useful in determining the severity of CAP, possibly due to medical advances [35]. Furthermore, a D- dimer level > 0.8 mg/L was found to be a risk factor for progressive CAP. The elevation of D-dimer in CAP is not fully understood. A meta-analysis indicated that elevated D-dimer levels were associated with CAP severity, pulmonary embolism (PE) occurrence and mortality [36].

The study had several limitations. First, it was a single-center retrospective study, and while internal verification was performed, the generalizability of the research findings to other hospitals remains uncertain. The presence of missing data resulted in the exclusion of variables with a significant number of missing values, which may be meaningful for the research results. Secondly, converting a numerical variable into a dichotomous variable through a straightforward transformation hinders the ability to obtain additional information. For example, it was not possible to ascertain the relationship between low body temperature and clinical deterioration or to establish a positive correlation between LDH levels and the risk of clinical deterioration in study. Furthermore, the study only provided a brief summary of imaging manifestations, which may have contributed to the absence of imaging features in the identified risk factors. Finally, the study did not conduct a detailed stratification of the degree of clinical deterioration, making it challenging to identify patients requiring urgent attention.

## Conclusion

The study identified the risk factors associated with the clinical deterioration of non-severe CAP and constructed a nomogram with robust predictive capabilities for clinical deterioration. This research provides valuable insights for the early identification and management of non-severe CAP patients at high risk of clinical deterioration.

### Electronic supplementary material

Below is the link to the electronic supplementary material.

Supplementary Material 1

## Data Availability

The datasets used and/or analysed during the current study are available from the corresponding author on reasonable request.

## Abbreviations

ACS: acute coronary syndrome
ALB: albumin
ALT: glutathione aminotransferase
ANC: absolute neutrophil value
AST: glutathione transaminase
AUC: area under curve
BMI: body mass index
BUN: Urea
CAP: community-acquired pneumonia
CK: creatine kinase
Cr: creatinine
CRP: C-reactive protein
DCA: decision curve analysis
HB: hemoglobin
HDL: high-density cholesterol
ICU: intensive care unit
LDH: lactate dehydrogenase
LDL: low-density cholesterol
LYM: absolute lymphocyte value
PCT: procalcitonin
PE: pulmonary embolism
PSI: pneumonia severity index
ROC: receiver operating characteristic
TBIL: total bilirubin
TC: total cholesterol
TG: triglycerides
UA: uric acid
WBC: white blood cell
