# Supplementary material for: Risk factors and predicting nomogram for the clinical deterioration of non-severe community-acquired pneumonia
Source: BMC Pulm Med. 2024 Jan 27;24:57. doi: 10.1186/s12890-023-02813-w (PMC10821265; doi:10.1186/s12890-023-02813-w)
Supplement: Supplementary file 1 — Supplementary Material 1 [file 12890_2023_2813_MOESM1_ESM.docx]

Table S1: clinical characteristics of non-clinical deterioration and clinical deterioration patients in all patients based on cutoff or median values

| Characteristics | Total (n=1632) | Non-Clinical Deterioration (n=1491) | Clinical Deterioration(n=141) | *P* value |
| --- | --- | --- | --- | --- |
| Sex, female n(%) | 675 (41) | 626 (42) | 49 (35) | 0.115 |
| Age ＞67 (year), n(%) | 443 (27) | 378 (25) | 65 (46) | < 0.001 |
| BMI ＞26.42 (kg/m^2^), n(%) | 232 (14) | 196 (13) | 36 (26) | < 0.001 |
| Hypertension, n (%) | 495 (30) | 441 (30) | 54 (38) | 0.04 |
| Diabetes, n (%) | 210 (13) | 187 (13) | 23 (16) | 0.252 |
| Cardiovascular disease^c^, n (%) | 155 (9) | 127 (9) | 28 (20) | < 0.001 |
| Liver disease, n (%) | 133 (8) | 124 (8) | 9 (6) | 0.521 |
| Gastrointestinal diseases, n (%) | 71 (4) | 67 (4) | 4 (3) | 0.48 |
| Chronic kidney disease, n (%) | 29 (2) | 26 (2) | 3 (2) | 0.734 |
| Cerebrovascular disease , n (%) | 66 (4) | 55 (4) | 11 (8) | 0.032 |
| Basic lung disease^b^, n (%) | 78 (5) | 67 (4) | 11 (8) | 0.12 |
| Pleural effusion, n (%) | 441 (27) | 385 (26) | 56 (40) | < 0.001 |
| Bilateral lung lesions, n (%) | 759 (47) | 679 (46) | 80 (57) | 0.014 |
| Time interval between onset and admission ＞2 (day), n (%) | 1482 (91) | 1353 (91) | 129 (91) | 0.889 |
| Duration of antibiotic use before admission ＞7 (day), n (%) | 81 (5) | 71 (5) | 10 (7) | 0.31 |
| Body temperature ＞38 (℃), n (%) | 299 (18) | 247 (17) | 52 (37) | < 0.001 |
| Systolic pressure ＞133 (mmHg), n (%) | 519 (32) | 460 (31) | 59 (42) | 0.01 |
| Diastolic pressure ＞84 (mmHg), n (%) | 381 (23) | 347 (23) | 34 (24) | 0.903 |
| Respiratory rate ＞21 (/min), n (%) | 52 (3) | 39 (3) | 13 (9) | < 0.001 |
| Pulse ＞95 (/min), n (%) | 541 (33) | 480 (32) | 61 (43) | 0.01 |
| SpO_2_ ＞97 (%), n (%) | 505 (31) | 473 (32) | 32 (23) | 0.034 |
| WBC ＞9.86 (×10^9^/L), n (%) | 410 (25) | 351 (24) | 59 (42) | < 0.001 |
| ANC ＞6.35 (×10^9^/L), n (%) | 556 (34) | 481 (32) | 75 (53) | < 0.001 |
| LYM ＞1.3 (×10^9^/L), n (%) | 815 (50) | 772 (52) | 43 (30) | < 0.001 |
| RBC ＞4.16 (×10^12^/L), n (%) | 812 (50) | 747 (50) | 65 (46) | 0.412 |
| HB ＞126 (g/L), n (%) | 796 (49) | 734 (49) | 62 (44) | 0.269 |
| PLT ＞366 (×10^9^/L), n (%) | 198 (12) | 177 (12) | 21 (15) | 0.36 |
| CRP ＞82.7 (mg/L), n (%) | 648 (40) | 557 (37) | 91 (65) | < 0.001 |
| PCT ＞0.078 (ng/ml), n (%) | 870 (53) | 759 (51) | 111 (79) | < 0.001 |
| TBIL ＞9 (μmol/L), n (%) | 662 (41) | 585 (39) | 77 (55) | < 0.001 |
| ALT ＞35 (U/L), n (%) | 519 (32) | 465 (31) | 54 (38) | 0.101 |
| AST ＞35 (U/L), n (%) | 476 (29) | 409 (27) | 67 (48) | < 0.001 |
| ALB ＞36 (g/L), n (%) | 832 (51) | 787 (53) | 45 (32) | < 0.001 |
| BUN ＞6.6 (mmol/L), n (%) | 275 (17) | 235 (16) | 40 (28) | < 0.001 |
| Cr ＞74 (μmol/L), n (%) | 554 (34) | 488 (33) | 66 (47) | 0.001 |
| eGFR ＞100.2, n (%) | 815 (50) | 768 (52) | 47 (33) | < 0.001 |
| UA ＞367 (μmol/L), n (%) | 199 (12) | 176 (12) | 23 (16) | 0.153 |
| K^+^ ＞4.41 (mmol/L), n (%) | 90 (6) | 81 (5) | 9 (6) | 0.78 |
| Na^+^ ＞139 (mmol/L), n (%) | 677 (41) | 638 (43) | 39 (28) | < 0.001 |
| CL^-^ ＞103 (mmol/L), n (%) | 694 (43) | 651 (44) | 43 (30) | 0.003 |
| TC ＞2.20 (mmol/L), n (%) | 1610 (99) | 1472 (99) | 138 (98) | 0.43 |
| TG ＞0.65 (mmol/L), n (%) | 1459 (89) | 1330 (89) | 129 (91) | 0.484 |
| HDL ＞0.92 (mmol/L), n (%) | 811 (50) | 756 (51) | 55 (39) | 0.01 |
| LDL ＞2.4 (mmol/L), n (%) | 810 (50) | 768 (52) | 42 (30) | < 0.001 |
| CK ＞120 (U/L), n (%) | 361 (22) | 312 (21) | 49 (35) | < 0.001 |
| LDH ＞234 (U/L), n (%) | 569 (35) | 486 (33) | 83 (59) | < 0.001 |
| D-Dimer ＞0.80 (mg/L), n (%) | 774 (47) | 668 (45) | 106 (75) | < 0.001 |

^b^: basic lung diseases including chronic bronchi, asthma, chronic obstructive pulmonary disease, silicosis in study. ^c^: cardiovascular disease including coronary atherosclerotic heart disease and atrial fibrillation. WBC: white blood cell. ANC: absolute neutrophil value. LYM: absolute lymphocyte value. HB: hemoglobin. CRP: C-reactive protein. PCT: procalcitonin. TBIL: total bilirubin. ALT: glutathione aminotransferase. AST: glutathione transaminase. ALB: albumin. BUN: Urea. Cr: creatinine. UA: uric acid. TC: total cholesterol. TG: triglycerides. HDL: high-density cholesterol. LDL: low-density cholesterol. CK: creatine kinase. LDH: lactate dehydrogenase.
